# The evolving landscape of molecular visualization

**DOI:** 10.1002/pro.70560

**Published:** 2026-04-11

**Authors:** Rachel Torrez, Hui Liu, Dillon Lee, Janet H. Iwasa

**Affiliations:** ^1^ Department of Biochemistry University of Utah Salt Lake City Utah USA

**Keywords:** animation, modeling, visualization

## Abstract

Molecular visualization plays a central role in structural biology, transforming data into representations that reveal how molecular form relates to function. Since the construction of the first protein models in the 1950s, visualization practices have evolved in tandem with experimental and computational advances, shaping both research and communication. Today's scientists rely on an expanding suite of digital tools to interpret structural, biophysical, and imaging data, while public repositories facilitate dissemination and education. Yet, as experimental methods capture ever more complex and dynamic molecular systems, the limitations of static visualizations have become apparent. Recent progress in animation, integrative modeling, and artificial intelligence offers new possibilities for representing molecular complexity and motion. This review traces the evolution of molecular visualization from physical models to dynamic, data‐integrated animations and explores how emerging technologies promise to make visualization not only a medium of communication but also a tool for scientific exploration.

## 1 INTRODUCTION

Across the life sciences, researchers work to analyze, interpret, and integrate a variety of data to create a mental model of biological processes. Molecular biologists often communicate these ideas to broad audiences through the construction of model figures that summarize and contextualize key findings. Model figures typically reflect a researcher's current working hypothesis and may integrate results from diverse experimental data (structural, biochemical, and kinetic) and different time scales. These figures can do more than simply illustrate a hypothesis; the process of creating a model figure can also prompt new questions and ideas and feed back into the research pipeline.

The field of molecular visualization has grown alongside the data it seeks to represent. Each advance in experimental resolution, from the first x‐ray structures to tomographic reconstructions of cellular environments, has spurred corresponding innovations in how data are depicted and explored (Johnson & Hertig, 2014; Li & Wei, 2024). Visualization is therefore both a mirror of technical progress and a driver of conceptual change. However, the back‐and‐forth between experimental data and tool development frequently leaves researchers waiting for the next advancement in molecular visualization to aid in further in‐depth analysis and communication. Understanding and anticipating the current and future needs of the scientific community is essential in the design and implementation of new molecular visualization tools.

With growing interest in understanding dynamic and crowded cellular environments, we envision that the next major advancement in molecular visualization tools should enable users to visualize complex and dynamic molecular and subcellular interactions. Since the rise of structural biology, we have increasingly been able to relate biochemical observations to protein function based on key insights from structural data. This integration of multiple types of data has led to major advances in our understanding of complex systems. For instance, structural biologists studying the nuclear pore complex were able to reveal new insights regarding the intricate architecture and function of this large multi‐domain complex by combining multiple experimental data types (Mosalaganti et al., 2022; Rout & Sali, 2019). This daunting task pushed the limits of many molecular visualization software and required researchers to utilize multiple tools in order to construct a complete molecular model. Supporting a larger number of researchers seeking to explore and visualize an ever‐growing list of complex molecular systems will potentially require a new class of software tools that can readily integrate data from diverse experimental modalities.

In this review, we highlight some of the growth in molecular visualization and how this field has evolved alongside experimental and technological advancements in structural biology. It also considers how emerging computational methods may transform the future of molecular storytelling.

## 2 FROM PLASTICINE MODELS TO RIBBON DIAGRAMS

In structural biology, the ability to view and manipulate a three‐dimensional protein model has long been critical for data analysis and communication. Nowadays, it is taken for granted that these 3 dimensional (3D) models are digital and can be readily viewed on a laptop or even a smartphone. Early pioneers in structural biology, however, lacked these modern conveniences and struggled to visualize molecular structures. Their hand‐crafted solutions have left a lasting mark on molecular visualization.

**FIGURE 1:**
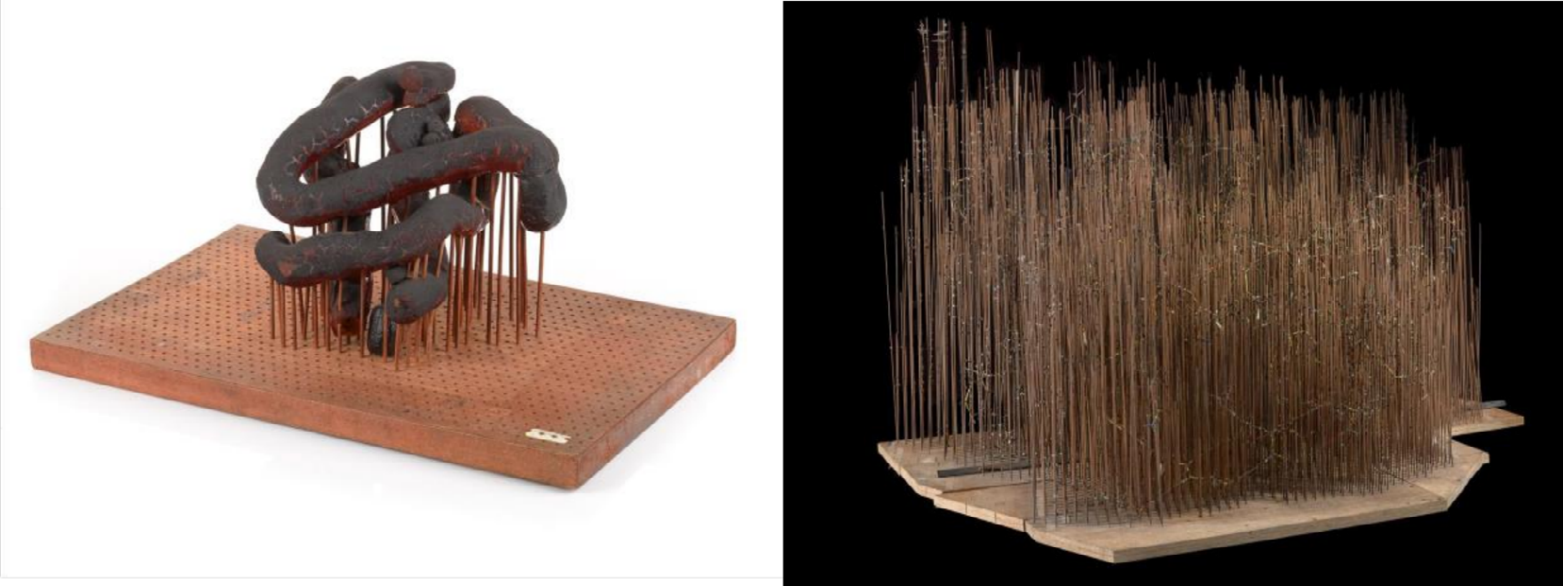
The first 3D protein models were physical models of myoglobin created by John Kendrew. Left: Sausage model; right: Forest of rods model (Credit: Science Museum Group).

During the late 1950s, the field of structural biology was beginning a period of rapid growth due to contributions by Linus Pauling, Robert Corey, Herman Branson, William L. Bragg, Max Perutz, and many others, but tools to quickly and easily analyze and share results were lacking (Bragg et al., 1950; Pauling et al., 1951; Perutz et al., 1960). The complex and often large datasets containing coordinate information presented a challenge, and structural biologists were forced to develop new methods to disseminate these structural results. One of many scientists leading the way for protein visualization and dissemination was John Kendrew, who, along with his research team, determined the first atomic‐level structure of myoglobin using x‐ray crystallography (Kendrew et al., 1958). This groundbreaking discovery paved the way for a new era in understanding protein structure, but at the time, analyzing x‐ray crystallography diffraction patterns was nearly impossible for non‐experts and extremely challenging at best for specialists. Additionally, as myoglobin was the first protein structure solved using x‐ray crystallography, there were no methods yet developed to display the structure of this size and complexity. Other space‐filling 3D models had been used previously to visualize structures for short polypeptide chains, DNA, and penicillin, in which the researchers focused on displaying the individual atoms, but applying a similar method to larger molecules was still a near impossible undertaking (Bragg et al., 1950; Hodgkin, 1949; Howard, 2003; Pauling et al., 1951; Watson & Crick, 1953). In spite of this, using the myoglobin density map as a guide, Kendrew created a plasticine model of the protein backbone suspended by sticks, and in doing so, created the first 3D protein model (Figure 1, *left*) (Kendrew et al., 1958; 1960). This model allowed for direct analysis of the overall protein structure and revealed the irregular shape of myoglobin. Photographs of the “sausage” model were later published for fellow researchers and crystallographers to analyze and interpret. While the model could “not be recommended on aesthetic ground” according to Kendrew (de Chadarevian, 2018), its effectiveness in communicating structural results was praised. There were shortcomings, however; the irregular structure of myoglobin made it difficult to describe using the sausage model, and it was clear that improvements needed to be made to better communicate and analyze atomic‐level details.

With this goal in mind, Kendrew and his team crafted a new 3D atomic model of myoglobin using steel rods, colored Meccano clips, and custom‐made skeletal model parts (Figure 1, *right*) (de Chadarevian, 2018). Often described as the “Forest of Rods”, this improved model was able to capture the complex intramolecular interactions occurring between amino acid residues within myoglobin. While the new “Forest of Rods” model allowed for greater detail to be shown, its complex rod design and intricate placement of amino acids made it more challenging to interpret by the untrained eye compared to the more simplified sausage model. This presented structural biologists with another challenge: How do we balance detail and understandability?

In 1961, the artist Irving Gies was commissioned by Scientific American to illustrate the structure of myoglobin (Dickerson, 1997; Gaber & Goodsell, 1997). This process took nearly 6 months to complete and required numerous sketches, studies, and photographs to transform the physical model into a comprehensible illustration. Gies would go on to illustrate numerous molecular structures. Over the course of his career, Gies adapted an increasingly abstract approach to visualizing protein structure, shifting away from atomic representations to something more abstract and artistic with a goal of creating understandable metaphors that illustrated the protein function alongside its structure. This artistic approach to visualizing protein structure inspired others to develop new conventions to communicate structural motifs in order to make more direct comparisons between proteins. One of the most significant examples was the development of the ribbon diagram representation pioneered by Jane Richardson (Kresge et al., 2011; Richardson, 1977). Prior to Richardson's innovation, there was no standardized way to visualize and compare protein structures. It was during this time that Jane Richardson began looking for patterns in folding preferences between protein structures. This led to the development of a simplified method to communicate a protein's overall topology while still maintaining key atomic information. Representing α‐helices as coiled ribbons and β‐sheets as arrows, Richardson developed the ribbon diagram style which omitted the atoms in the 3D structure and instead focused on the backbone of the structure. This allowed for easier comparison between proteins and successfully communicated the 3D relationships within the protein structure on a 2D page. In 1981, Richardson's hand‐drawn ribbon diagrams were published in the journal *Advances in Protein Chemistry* in which she used this visualization style to showcase and classify the structure of 75 proteins (Kresge et al., 2011). This achievement revolutionized the way in which protein structures were represented and is still used today.

While these early molecular visualization methods provided researchers with a way to analyze and interpret protein structures, their accuracy and reproducibility were limited. Since many of these 3D protein models were created by hand, their reliability was dependent on the artist's ability to correctly interpret and communicate the complex and intricate structural data. Additionally, the time‐consuming nature of hand‐drawn models made it nearly impossible to match the rapid influx of new protein structures.

## 3 THE GROWTH DIGITAL VISUALIZATION TOOLS

As the field of computer graphics advanced alongside structural biology, molecular visualization software moved to center stage. The foundations of this field can trace back to Cyrus Levinthal, who recognized the potential of computers for building and exploring macromolecular structures. In his seminal Scientific American article, “Molecular model‐building by computer” (Levinthal, 1966), he envisioned interactive graphics that allowed scientists to examine complex conformations beyond the reach of physical models, establishing concepts that remain central to molecular visualization today. Over subsequent decades, these ideas evolved with advances in graphics and structural methods, leading to modern software that integrates analysis and visualization in user‐friendly platforms. Overviews of representative molecular visualization tools can be found in O'Donoghue et al. (2010) and Li and Wei (2024).

While early software focused primarily on developing specific analysis tools to meet research needs, packages such as University of California, San Francisco Chimera and PyMol, which were developed in the late 1990s and early 2000s, balanced data analysis and visual communication needs, allowing users to readily create publication‐ready images (Goddard et al., 2018; Li & Wei, 2024; Martinez et al., 2019; Pettersen et al., 2021). These softwares offer a plethora of tools to visually analyze structures, such as molecular interaction analysis, structure superposition, binding site detection, and electrostatic mapping, in addition to providing users with options to adjust graphical elements such as lighting, color, representation, and transparency to design and create high‐quality figures. By combining research and visualization applications into a singular tool, the analysis, interpretation, and dissemination of results can be streamlined and standardized.

The development of digital molecular models accompanied the establishment of web‐based databases for sharing structural data. The primary structural database used globally is the Protein Data Bank (PDB) (Burley et al., 2017; Rose et al., 2017). Since its establishment in 1971, the PDB contains over 200,000 structures, ranging from single chain proteins and nucleic acids to complex molecular machines. While its initial purpose was to serve as a publicly available archive for structural data, today it is used for a variety of purposes beyond just research (Rose et al., 2017). For instance, the Molecule of the Month series, first started in 2000 by structural biologist and artist David Goodsell, began utilizing molecular visualization tools to tell stories about PDB structures (Goodsell et al., 2020). These articles often included visually informative illustrations of protein structures alongside text that described the protein functions and diverse roles within cells. The development of these articles eventually led to the official release of the educational online portal PDB‐101 in 2011 (Zardecki et al., 2022).

In parallel with the expansion of experimentally determined structures, new data resources have emerged to support increasingly complex structural models and imaging modalities (Burley et al., 2019). The PDB‐Integrative/Hybrid Methods (PDB‐IHM) archive extends the PDB framework to enable deposition of structural models derived from integrative or hybrid approaches that combine heterogeneous data types across scales (Vallat et al., 2025). In addition, databases such as the Electron Microscopy Data Bank (EMDB) and the Chan Zuckerberg Initiative CryoET Data Portal support the deposition and dissemination of raw, segmented, and annotated cryo–electron tomography datasets, providing essential infrastructure for multiscale structural modeling and visualization of macromolecular organization in cellular contexts (Ermel et al., 2024; The wwPDB Consortium, 2024). Collectively, these online databases function as comprehensive resources for both researchers and educators, allowing for a more direct line of communication and public sharing of scientific findings.

## 4 VISUALIZING CROWDS

Contemporary structural biology confronts systems far more complex than the isolated molecules of early crystallography. Cryo‐electron microscopy (cryo‐EM) can capture large macromolecular assemblies in diverse states, and cryo‐electron tomography (cryo‐ET) reveals complexes within their native crowded cellular contexts (Carugo and Djinović‐Carugo, 2023; Schwalbe et al., 2024). A single static 3D model can no longer capture the depth of information that most researchers would like to convey. New tools are needed to visualize complex and dynamic molecular models, but depicting this complexity challenges both perception and computation.

**FIGURE 2:**
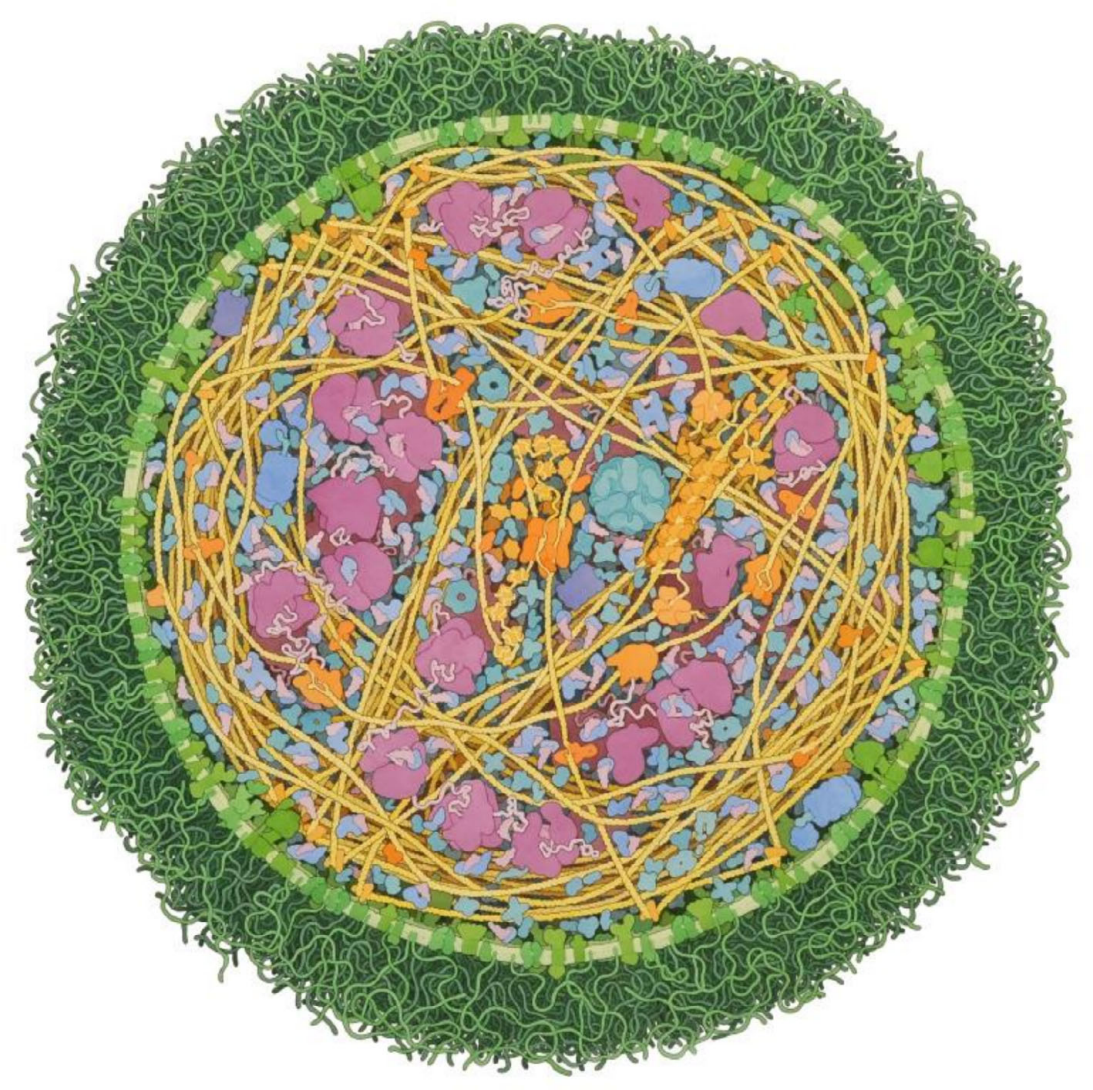
Illustration of *Mycoplasma mycoides* by David Goodsell.

Perhaps some of the best examples of visualizing molecular complexity are actually hand‐painted. David Goodsell, in addition to his contributions to the Research Collaboratory for Structural Bioinformatics PDB's Molecule of the Month, is well known for his unique style of visualizing crowded molecular landscapes (Figure 2; Goodsell et al., 2018). By integrating biochemical and structural data into colorful molecular scenes, his work has inspired new tools such as CellPack (Johnson et al., 2015), CellPaint (Gardner et al., 2018), and CellWalk (Davison, n.d.) (https://cellwalk.ca), pointing the way for further development in molecular visualization tools that aim to accommodate the changing needs of researchers and science communicators.

Alongside CellPack and CellPaint, a growing body of work has been addressing the visualization challenges posed by large, crowded, and multiscale biological systems. Earlier tools summarized by O'Donoghue et al. (2010), together with advances in web‐based molecular graphics, have greatly expanded access to molecular visualization. WebGL‐enabled viewers such as NGL Viewer (Rose et al., 2018), Mol* (Sehnal et al., 2021), and related platforms support interactive, high‐quality rendering directly in web browsers, facilitating rapid inspection and dissemination of crowded and multiscale biological structures. To tackle computational challenges, recent research has introduced algorithmic strategies for managing visual complexity, including scalable rendering approaches such as virtual instancing and adaptive level‐of‐detail methods (Alharbi et al., 2025). Together, these approaches reflect a shift toward visualization frameworks that integrate structural detail across molecular, cellular, and mesoscale contexts.

## 5 VISUALIZING DYNAMICS

Structural techniques like x‐ray crystallography have traditionally provided a frozen snapshot of a molecular complex in a discrete conformation. In the cell, however, we know that many proteins exhibit conformational and compositional heterogeneity that is closely linked to their function (Orellana, 2019). Recent advances in structural biology have shifted the focus from the determination of static structures to dynamic visualizations of conformational ensembles (Grandori, 2023). Techniques such as cryo‐EM and cryo‐ET now allow macromolecular structures to be visualized at near‐atomic resolution in their near‐native environment (Berger et al., 2023; Chari & Stark, 2023). These approaches have made it possible to capture protein complexes in multiple conformational states, providing unprecedented insights into molecular flexibility and compositional heterogeneity that were previously inaccessible.

In parallel, computational biology has introduced powerful tools for analyzing and interpreting the massive datasets generated by cryo‐EM and cryo‐ET studies. For instance, 3D variability analysis in CryoSPARC enables the exploration of continuous conformational changes within single‐particle cryo‐EM data (Punjani & Fleet, 2021). CryoDRGN is designed to reconstruct continuous 3D density maps from single‐particle cryo‐EM data, enabling visualization and analysis of per‐particle structural heterogeneity and molecular motions (Rangan et al., 2024). Its extension, CryoDRGN‐ET, also leverages deep learning but is further developed to analyze structural heterogeneity in cryo‐ET subtomogram data. Additional methods, including HEMNMA‐3D (Harmonic ENM‐based Normal Mode Analysis in 3D), MDSPACE (Molecular Dynamics simulation for Single Particle Analysis of Continuous Conformational hEterogeneity), and MDTOMO (Molecular Dynamics‐based Tomography Analysis), apply molecular dynamics‐based simulations to extract continuous conformational landscapes from cryo‐ET data (Harastani et al., 2021; Vuillemot et al., 2023, 2024). These methods enable the characterization of low‐frequency motions and flexible domain rearrangements that are often obscured in conventional discrete reconstructions, providing a more complete picture of the dynamic behavior of macromolecular complexes.

Despite these advances, existing methodologies primarily provide static representations of dynamic molecular systems. Although multiple conformations can be reconstructed from cryo‐EM and cryo‐ET data, these remain discrete snapshots sampled along a continuous conformational landscape. The transitions between states—how proteins shift, fold, or assemble in real time—remain largely inferred rather than directly observed (Mäeots & Enchev, 2022). This limitation has driven increasing interest in visualization and animation techniques that can bridge the gap between discrete structural reconstructions and continuous conformational dynamics.

Molecular visualization software such as PyMOL (Delano, 2002) and Chimera/ChimeraX (Goddard et al., 2018; Pettersen et al., 2021) facilitate the generation of morphs between known conformations, producing continuous trajectories that help illustrate possible transition pathways. PyMOL also supports loading trajectories or multi‐state models and viewing them as animations. However, such approaches typically rely on linear interpolation and thus cannot fully represent the stochastic, multidimensional nature of molecular motion. Consequently, while these tools enhance interpretability and accessibility, they remain limited in their ability to demonstrate the true complexity of protein dynamics.

Advancements in molecular modeling and AI have the potential to make significant contributions to how we visualize dynamic molecular processes. For example, molecular dynamics simulations, while limited in time and length scales due to computational cost, can be combined with methods such as Monte Carlo simulations to create simulations of molecular systems efficiently over longer timescales (Bernardi et al., 2015; Mohr et al., 2024). Molecular dynamics simulations are also increasingly integrated with cryo‐EM, nuclear magnetic resonance spectroscopy, and x‐ray scattering to characterize conformational heterogeneity and bridge static structural data with dynamic molecular movements (Son et al., 2024; Zadorozhnyi et al., 2023). In addition, agent‐based modeling provides a powerful framework for exploring complex biological systems by simulating the behavior of individual molecular or cellular components according to defined rules (Riggi & Iwasa, 2025). These models can allow researchers to study emergent phenomena that arise from local interactions, offering insight into how collective behaviors and dynamic structures form within cells.

Recent advances in artificial intelligence are further expanding the possibilities for predictive molecular modeling. Tools such as Alphafold (Abramson et al., 2024), AlphaFold‐Multimer (Evans et al. 2021), and related AI‐based systems can make predictions of how proteins interact to form complexes, potentially providing new insights into our understanding of molecular assembly and disassembly. As these predictive models improve, they could serve as foundations for generating semi‐automated molecular animations that visualize the formation, dynamics, and regulation of macromolecular complexes.

## 6 INTEGRATING DATA AND VISUALIZING HYPOTHESES

Biological processes typically involve numerous molecules and interactions spanning multiple spatial and temporal scales. As a result, protein dynamics encompass not only conformational changes at the molecular level, but also large‐scale structural rearrangements and intermolecular interactions at the cellular scale (Grandori, 2023). To achieve a comprehensive understanding of such complex dynamics and how protein structures are linked to their cellular functions, structural data need to be integrated with complementary temporal and contextual information. Bringing together such diverse and often incomplete datasets into a coherent narrative remains a major challenge in modern biology.

Molecular animation offers one solution to representing complex biological processes in ways that align closely with researchers' mental models (Iwasa, 2015). By integrating and synthesizing diverse types of experimental and computational data, animators can construct coherent molecular or cellular “stories” that illustrate dynamic mechanisms and spatial relationships. These visualizations are often highly intuitive and accessible, allowing both specialists and non‐specialists to grasp intricate molecular events and facilitating communication across disciplines.

**FIGURE 3:**
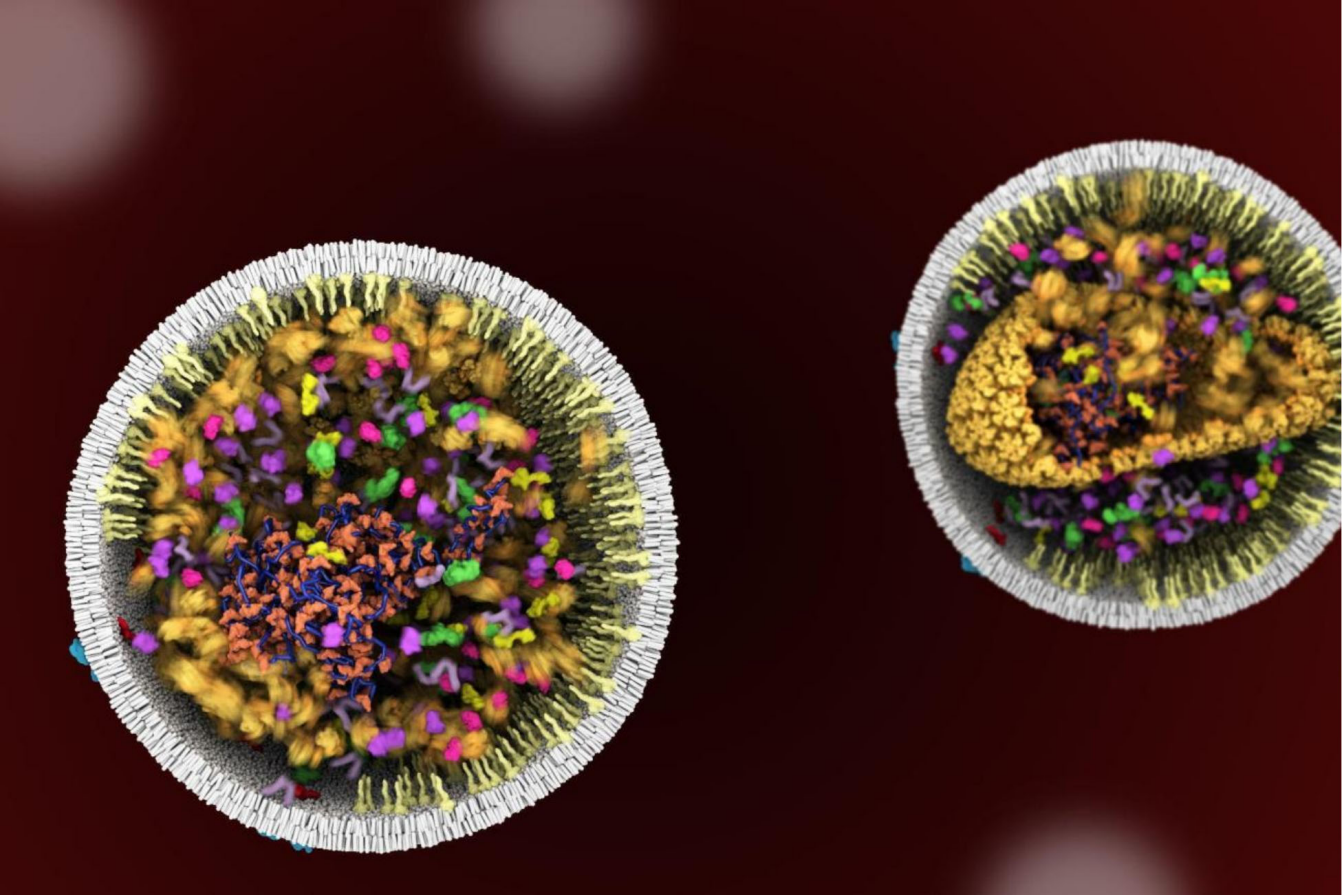
Still image from 3D animation depicting the maturation step of the Human Immunodeficiency Virus life cycle.

Over the past 20 years, the use of 3D animation to depict biological processes has grown dramatically. In research, animations are now widely used to synthesize, contextualize, and communicate both new discoveries and existing knowledge about molecular and cellular mechanisms (Iwasa, 2022). For example, our lab has created numerous animations using 3D animation software, including animations illustrating virus life cycles and protein complex assembly within a cellular context. Although 3D animation software was originally developed for the gaming and film industries, they can be highly effective for constructing dynamic protein models and animating their interactions. Our lab uses Autodesk Maya and Blender to build dynamic protein models, taking advantage of their ability to import and animate molecular structures derived from different sources, such as PDB (Burley et al., 2017) and the AlphaFold Protein Structure Database (Abramson et al., 2024). This approach allows us to combine structures from different PDB files, reconstruct missing regions using AlphaFold‐predicted structures, add flexible linkers between rigid domains, and contextualize molecular processes within a modeled cellular space (Figure 3).

Many of our lab's animation projects focus on visualizing protein dynamics at the mesoscale or cellular level. These visualizations generally depict intermolecular interactions and large‐scale molecular movements that, until recently, were difficult to observe or capture experimentally (Orellana, 2019). These dynamic processes often involve multiple molecules moving in crowded environments over varying spatial and temporal scales, making them also inherently challenging to visualize. The limited availability of dynamic data also made the process of creating animations challenging. In recent years, however, a variety of experimental and computational methods have been developed that provide increasingly detailed insights into molecular dynamics. Many of our recent animations have already benefited from the increasing availability of dynamic data. For example, we can now combine high‐resolution structural models with complementary dynamic information, such as spatiotemporal tracking from advanced light microscopy techniques or coarse‐grained molecular dynamics simulation data (Riggi et al., 2024).

The emergence of new data types and sources has become a major driving force in the advancement of animation workflows and visualization tools. This progress brings with it the challenge of developing methods that can effectively harness these datasets to construct dynamic models that are accurate both structurally and temporally. As dynamic data become increasingly accessible, new techniques can be developed to systematically incorporate this data into animated trajectories. These data‐driven animations have the potential to yield important mechanistic and quantitative insights.

Molecular animation has numerous advantages but also suffers from several notable limitations. Even for experienced animators, creating a high‐quality animation is time‐intensive, limiting the scalability of this approach for iterative research. Additionally, scene files and underlying data are rarely shared, reducing transparency (Alharbi et al., 2025). From a viewer's perspective, it is typically impossible to discern whether an animation is grounded in empirical data or reflects speculative interpretation. Finally, molecular animations cannot directly test hypotheses in the way simulations can; they lack the quantitative rigor and computational framework necessary for data‐driven hypothesis testing.

To address the growing need for accessible molecular animation tools, a number of plugins have been developed over the years for different platforms, including Molecular Maya, BioBlender (Andrei et al., 2012), and CellPack (Johnson et al., 2015). More recently, Molecular Nodes has been developed for the open‐source animation software Blender (Johnston et al., 2025). In addition, our group is currently working on new Blender‐based molecular animation tools, as well as developing workflows for sharing and annotating animations.

## 7 PEEKING OVER THE HORIZON

The trajectory of molecular visualization has always mirrored the growth of structural biology, starting from hand‐built models that showed the architecture of proteins to digital platforms that integrate structural, biophysical, and imaging data into compelling visual narratives. Each generation of tools has expanded what can be seen and imagined.

Today, molecular visualization stands at a crossroads. Structural techniques can now capture unprecedented molecular complexity, while AI and integrative modeling generate predictive insights across scales. To harness these advances, visualization must evolve toward open, interoperable ecosystems that support both scientific exploration and transparent communication.

Future progress will depend on developing community standards for representing uncertainty, documenting data provenance, and sharing interactive visualizations alongside publications. Integrating animation, simulation, and AI within reproducible workflows will transform visualization from a final step in the dissemination pipeline into an active component of discovery.

## AUTHOR CONTRIBUTIONS

**Rachel Torrez:** Conceptualization; writing – original draft; writing – review and editing. **Hui Liu:** Conceptualization; writing – original draft; writing – review and editing. **Dillon Lee:** Conceptualization; writing – original draft; writing – review and editing. **Janet H. Iwasa:** Writing – review and editing; conceptualization; writing – original draft.

## CONFLICT OF INTEREST STATEMENT

None of the authors have a conflict of interest to disclose.

## Data Availability

Data sharing not applicable to this article as no datasets were generated or analysed during the current study.
